# Interplay between SIN3A and STAT3 Mediates Chromatin Conformational Changes and GFAP Expression during Cellular Differentiation

**DOI:** 10.1371/journal.pone.0022018

**Published:** 2011-07-11

**Authors:** Pei-Yi Cheng, Yu-Ping Lin, Ya-Ling Chen, Yi-Ching Lee, Chia-Chen Tai, Yi-Ting Wang, Yu-Ju Chen, Cheng-Fu Kao, John Yu

**Affiliations:** 1 Graduate Institute of Life Sciences, National Defense Medical Center, Taipei, Taiwan; 2 Genomics Research Center, Academia Sinica, Taipei, Taiwan; 3 Institute of Cellular and Organismic Biology, Academia Sinica, Taipei, Taiwan; 4 Institute of Biomedical Science, Academia Sinica, Taipei, Taiwan; 5 Institute of Chemistry, Academia Sinica, Taipei, Taiwan; Louisiana State University Health Sciences Center, United States of America

## Abstract

**Background:**

Neurons and astrocytes are generated from common neural precursors, yet neurogenesis precedes astrocyte formation during embryogenesis. The mechanisms of neural development underlying suppression and de-suppression of differentiation- related genes for cell fate specifications are not well understood.

**Methodology/Principal Findings:**

By using an *in vitro* system in which NTera-2 cells were induced to differentiate into an astrocyte-like lineage, we revealed a novel role for Sin3A in maintaining the suppression of GFAP in NTera-2 cells. Sin3A coupled with MeCP2 bound to the GFAP promoter and their occupancies were correlated with repression of GFAP transcription. The repression by Sin3A and MeCP2 may be an essential mechanism underlying the inhibition of cell differentiation. Upon commitment toward an astrocyte-like lineage, Sin3A- MeCP2 departed from the promoter and activated STAT3 simultaneously bound to the promoter and exon 1 of GFAP; meanwhile, olig2 was exported from nuclei to the cytoplasm. This suggested that a three-dimensional or higher-order structure was provoked by STAT3 binding between the promoter and proximal coding regions. STAT3 then recruited CBP/p300 to exon 1 and targeted the promoter for histone H3K9 and H3K14 acetylation. The CBP/p300-mediated histone modification further facilitates chromatin remodeling, thereby enhancing H3K4 trimethylation and recruitment of RNA polymerase II to activate GFAP gene transcription.

**Conclusions/Significance:**

These results provide evidence that exchange of repressor and activator complexes and epigenetic modifications are critical strategies for cellular differentiation and lineage-specific gene expression.

## Introduction

During embryonic development, the generation of three major neural cell types (neurons, astrocytes, and oligodendrocytes) in the central nervous system (CNS) sequentially occurs, whereby almost all neurons are generated before the appearance of glial cells [1], [2]. Recent findings demonstrated that glial cells are important in critical neuronal maturation processes such as axonal pathfinding, synapse formation, neurotransmitter transport, metabolic functions, and the response to CNS injury [3]–[6]. Although rodent brain cultures and neuronal and glial cell lines have provided us with important information about the structure and function of the mammalian CNS, we have scanty understanding of astrocytic differentiation.

There has been longstanding interest in understanding how the process by which progenitors differentiate into different cell types is regulated. In a mouse model, the fate of progenitors in the developing brain is believed to be determined by external cues that involve various types of cytokines and internal cellular programs. External cues such as bone morphogenetic proteins, leukemia inhibitory factor, ciliary neurotrophic factor, Notch-Delta, and basic fibroblast growth factor promote astrocytic differentiation [7]–[13], and most of these factors influence the essential astrogliogenic Janus kinase-signal transducer and activator of transcription pathway [14]–[17]. A molecular basis for the cooperative action between these families of cytokines involves the formation of a STAT3-Smad1 complex with the coactivator, p300/CBP, that initiates astrocyte-specific gene expression [15], [18]–[20].

Intrinsic programs regulating cell fate determination of progenitors include epigenetic modifications such as DNA methylation and chromatin remodeling. Methylation of the STAT-binding element within the glial fibrillary acidic protein (GFAP) promoter in mice was shown to inhibit the association of activated STATs with the glial promoter, thereby repressing transcription of the GFAP gene [10], [16], [21]. Furthermore, conditional deletion of the maintenance DNA methyltransferase I from neural progenitor cells (NPCs) suggests that DNA methylation regulates the timing and magnitude of astrogliogenesis [22]. Another class of epigenetic modifications was found from FGF2, which regulates the ability of ciliary neurotrophic factor to enhance astrocyte differentiation by inducing H3 Lys4 dimethylation and suppressing H3 Lys9 dimethylation at the STAT3-binding site, resulting in access of the STAT/CBP complex to the GFAP promoter and activation of GFAP expression [16]. Those reports highlight the diverse epigenetic mechanisms that control lineage-specific gene expression; however, it remains unclear how the interplay among DNA methylation, transcriptional repressors or activators, and histone modifications contributes to regulation of the processes.

In this study, we used a human embryonal carcinoma cell line, NTera-2, to develop a model that induces the differentiation of these cells into an astrocyte-like lineage. NTera-2 is derived from a human teratocarcinoma which shares many characteristics of neuroepithelial precursor cells and is widely used as a tool to study the early development of the human CNS and identify new genes involved in neurogenesis [23]–[25]. We also used this system to investigate the mechanisms underlying GFAP activation. We identified components of the Sin3A-HDAC complex coupled with MeCP2 present at the GFAP promoter under undifferentiated conditions. Upon differentiation, the promoter underwent a conformational change triggered by STAT3 binding, which contributes to CBP/p300-mediated histone modification and assembly of a transcription preinitiation complex, resulting in GFAP gene activation.

## Materials and Methods

### Cell lines and reagents

NTera-2, 3T3, and 293T cells were obtained from the American Type Culture Collection (Rockville, MD, USA). Cells were cultured in Dulbecco's modified Eagle medium (DMEM; Invitrogen, Durham, NC, USA) containing 10% fetal bovine serum (FBS; JRH Bioscience, Lenexa, KS, USA), 0.2 mM GlutaMax-1 (Invitrogen), and penicillin/streptomycin (100 units/mL; Invitrogen). Cells were split every 2 days in a 0.5% trypsin-EDTA solution and maintained until used.

### Design of the Oct-4 RNAi vector

An oligonucleotide containing a stem-loop structure targeting the *Oct-4* gene was designed using the RNAi program (http://athena.bioc.uvic.ca/). The targeted sequence was GCGAACCAGTATCGAGAAC in the *Oct-4/POU5F1* gene (515∼534 nt; accession no. NM_203289). This sequence was first cloned into a pBS/U6 vector. Then the U6-RNAi cassette was subcloned into a lentiviral vector, pFUGW [26], and the sequences were verified by DNA sequencing.

### Lentiviral production and NTera-2 cell transduction

Lentiviral vectors were produced by transient co-transfection of pCMVΔR8.9 (10 µg), VSV-G (pMD.G; 10 µg), and the lentiviral vector (pFUGW; 10 µg) into 293T cells. Viral supernatants were concentrated by ultracentrifugation to produce viruses with titers of 1×10^8^ infection units/mL. Multiplicities of infection of 10 and 25 were used to infect NTera-2 cells in the presence of 8 µg/mL polybrene (Sigma, St. Louis, MO, USA). These transduced cells expressed green fluorescent protein (GFP) and were analyzed by fluorescence-activated cell sorting (FACS).

### Cell-cycle analysis

Cells were resuspended in PBS and fixed with ethanol overnight at −20°C. Cells were then resuspended in PBS and treated with 100 µg/ml of ribonuclease A (bovine pancreas; Sigma), 0.1% Triton-X100, and 40 µg/ml propidium iodide (PI) for 30 min at 37°C. Cell cycles were detected with FACSCalibur, and analyzed by the ModFit LT program (Verity Software House, Topsham, ME, USA).

### Reverse-transcription polymerase chain reaction (RT-PCR)

Total RNA was extracted from NTera-2 cells using the Qiagen RNeasy Mini kit following the manufacturer's protocol (Qiagen, Valencia, CA, USA). The target RNA was amplified by a one-step RT-PCR kit (GMbiolab, Taichung, Taiwan). Forward and reverse primers were as follows: GFAP (forward), 5′-GTGGGCAGGTGGGAGCTTGATTCT-3′ and (reverse), 5′-CTGGGGCGGCCTGGTATGACA-3′ [27]; and internal control β-actin (forward), 5′-TGGAATCCTGTGGCATCCATGAAAC-3′ and (reverse), 5′-TAAAACGCAGCTCAGTAACAGTCCG-3′. The RT-PCR consisted of two programs. First was complementary cDNA synthesis: one cycle at 50°C for 30 min and one cycle at 94°C for 2 min. The other was second-strand cDNA synthesis and the PCR consisted of 35 cycles at 94°C for 30 s, 59°C for 30 s, and 72°C for 45 s; followed by a final extension step at 72°C for 10 min.

### Western blotting

At different times after viral infection, cells were lysed in lysis buffer (4 M urea, 1 mM MgCl_2_, 50 mM HEPES, and 100 U benezonase/10^7^ cells). Lysates containing the equivalent of 2×10^5^ cells per lane were separated by electrophoresis on 10% polyacrylamide gels and electrotransferred onto polyvinylidene difluoride membranes overnight. The membrane was blocked with 5% fat-free milk in a TBST solution for 1 h and then probed separately with a mouse monoclonal antibody (mAb) against Oct-4 (Santa-Cruz Biotechnology, Santa Cruz, CA, USA) at a concentration of 0.2 µg/mL, 1∶1000 of an anti-GFAP rabbit polyclonal antibody (pAb; Chemicon International, Temecula, CA, USA), and 1∶500 of an anti-glutamine synthetase mouse mAb (Chemicon International). Mouse β-actin was used as an internal control (Sigma). Horseradish peroxidase (HRP)-conjugated anti-mouse immunoglobulin G (IgG) and anti-rabbit IgG (Sigma) were used as the secondary antibodies. Signals were detected using an enhanced chemiluminescence (ECL) kit (Perkin Elmer, Wellesley, MA, USA).

### Immunoprecipitation

Mock-, vector-, and RNAi-treated cells were solubilized in NP40 lysis buffer (10 mM Tris at pH 7.5, 150 mM NaCl, 0.5% NP40, 5 mM EDTA, 5 mg/ml aprotinine, and 3 mM pAPMSF) and then centrifuged at 12,000 rpm for 10 min at 4°C. The lysates were subjected to immunoprecipitation with an anti-STAT3 antibody (Santa Cruz Biotechnology). Then, these immunocomplexes were analyzed as described for the Western blot assay. These immunocomplexes were detected by an anti-p300 antibody (Santa-Cruz Biotechnology).

### Immunocytochemistry

NTera-2 cells were grown on 12-well plates. These cells were washed with phosphate-buffered saline (PBS) and fixed with 3% paraformaldehyde. Then, cells were permeabilized in 0.25% Triton X-100 and incubated with the following primary antibodies overnight at 4°C: anti-Oct-4 rabbit Ab (1∶200), anti-Pax6 mouse Ab (1∶50), anti-Olig2 rabbit Ab (1∶50; Santa-Cruz Biotechnology), anti-nestin mouse Ab (1∶100), anti-A2B5 mouse Ab (1∶200), anti-glial fibrillary acidic protein rabbit Ab (1∶1000), anti-MAP2 rabbit Ab (1∶1000), anti-glutamine synthetase mouse Ab (1∶500; Chemicon International). The next day, cells were washed with PBS and secondary antibodies of 1∶500 of goat-mouse-594 (Molecular Probe, Carlsbad, CA, USA), 1∶500 of goat-rabbit-cy3, and 1∶200 of goat-mouse-cy3-IgM (Jackson ImmunoResearch, Suffolk, UK) which were used accordingly for 1 h at 37°C. Cells were then washed with PBS and counter-stained with Hoechst for 10 min. Cells were detected under florescence microscopy.

### Label-free quantitation method

Preparation of Protein Extracts: Cell pellets were first washed three times with PBS and lysed in lysis buffer (0.25 M Tris-HCl at pH 6.8, 1% SDS). Protein concentration was obtained by BCA™ protein assay. Cell lysate was then dried and stored in −30°C.

Gel-assisted Digestion [28]: Acrylamide/bisacrylamide (40%, 29∶1), 10% APS, and TEMED were then applied to protein solution to polymerize as a gel directly in the eppendorf. The gel was cut into small pieces and washed several times with TEABC containing 50% ACN. The gel samples were further dehydrated with ACN and the completely dried by vacuum centrifugation. Proteolytic digestion was then performed with trypsin (protein:trypsin = 50∶1, g/g) in 25 mM TEABC with incubation overnight at 37°C and peptides were extracted by 5% FA in 50% ACN, dried in a SpeedVac and stored in −30°C.

Immobilized Metal Affinity Chromatography (IMAC) [29]: The IMAC column was first capping one end with a 0.5 µm frit disk enclosed in stainless steel column-end fitting. The Ni-NTA resin was extracted from spin column and packed into a 5-cm microcolumn (500 µm id PEEK column). Automatic purification of phosphopeptides was performed using the IMAC microcolumn connected with autosampler and HP1100 solvent delivery system with a flow rate 13 µl/min. First, the Ni2+ ions were removed with 100 µl EDTA (50 mM) in NaCl (1 M). Then the IMAC column was activated with 100 µl FeCl3 (0.2 M) and equilibrated with loading buffer for 30 min before sample loading. For optimization of the phosphopeptide enrichment, the loading/condition buffer (designated as loading buffer) was 6% (v/v) AA and the pH was adjusted to 3.0 with NaOH (0.1 M at pH 12.8). The peptide samples from trypsin digestion were reconstituted in loading buffer and loaded into the IMAC column that had been equilibrated with the same loading buffer for 20 min. Then the unbound peptides were removed with 100 µl washing solution consisting of 75% (v/v) loading buffer and 25% (v/v) ACN, followed by equilibration with loading buffer for 15 min. Finally, the bound peptides were eluted from the IMAC column with 100 µl NH4H2PO4 (200 mM at pH 4.4). Eluted peptide samples were dried under vacuum and reconstituted in 0.1% (v/v) FA for LC- MS/MS analysis.

LC-MS/MS Analysis: Purified phosphopeptide samples were reconstituted in 4 µl buffer A (0.1% FA in H_2_O) and analyzed by LC-Q-TOF MS (Waters Q-TOFTM Premier from Waters Corp). For LC-MS/MS analysis by Waters Q-TOFTM Premier, samples were injected into a 2 cm×180 µm capillary trap column and separated by 20 cm×75 mm Waters1 ACQUITYTM 1.7 mm BEH C18 column using a nanoACQUITY Ultra Performance LCTM system (Waters Corp). The column was maintained at 35°C and eluted with a linear gradient of 0–80% buffer B (0.1% FA in ACN) for 80, 120, 210 and 270 min. MS was operated in ESI positive V mode with a resolving power of 10,000. NanoLockSpray source was used for accurate mass measurement and the lock mass channel was sampled every 30 s. The mass spectrometer was calibrated with a synthetic human [Glu1]-Fibrinopeptide B solution (1 pmol/µl; Sigma) delivered through the NanoLockSpray source. Data acquisition was operated in the data directed analysis (DDA). The method included a full MS scan (m/z 400–1600, 0.6 s) and 3 MS/MS (m/z 100–1990, 1.2 s each scan) sequentially on the most three intense ions present in the full scan mass spectrum.

Database Search, Protein Identification and Quantitation: Raw data were processed using Mascot Distiller v 2.1.1.0 (Matrix science). The resulting MS/MS dataset was exported to *mzdata data file format. We performed the peptide identification and assignment of partial post-translational modifications using an in-house version of Mascot v. 2.2 (Matrix science). The datasets were searched against International Protein Index (IPI_human v. 3.29, 68161 sequences) using the following constraints: only tryptic peptides with up to two missed cleavage sites were allowed; 0.3-Da mass tolerances for MS and 0.1-Da mass tolerances for MS/MS fragment ions. Phosphorylation (STY), oxidation (M) was specified as variable modifications. Only unique peptide with scores higher than 25 was confidently assigned. When unique peptides were identified to multiple members of a protein family, proteins having the highest sequence coverage were selected from the Mascot search output result. To evaluate the protein identification false-positive rate, we repeated the searches using identical search parameters and validation criteria against a random database (from the 68161 sequence). Relative quantification of peptides was performed by IDEAL-Q software [30], [31].

### DNA methylation analysis

Genomic DNA sodium bisulfite conversion was performed using an EZ-96 DNA methylation kit (Zymo Research, Orange, CA, USA). The manufacture's protocol was followed using 1 µg of genomic DNA and an alternative conversion protocol (two-temperature DNA denaturation).

Seqqunom's (San Diego, CA, USA) MassARRAY platform was used to perform the quantitative methylation analysis. This system utilizes MALDI-TOF mass spectrometry in combination with RNA base-specific cleavage (MassCLEAVE kit). A detectable pattern is then analyzed to determine the methylation status. PCR primers were designed using EpiDesigner (Sequenom). Amplicons were designed to cover the CpG sites from an approximately 4500-bp region upstream of the GFAP transcription start site to the intron 1 region. For each reverse primer, an additional T7 promoter tag for in vivo transcription was added, as well as a 10-mer tag on the forward primer to adjust for differences in melting temperatures. The PCRs were carried out in a 5-µl format with 10 ng/µl bisulfite-treated DNA, 0.2 units of HotStart *Taq* DNA polymerase (Qiagen), 1× supplied HotStart buffer, and 200 µM PCR primers. Amplification of the PCR was as follows: preactivation at 95°C for 15 min, 45 cycles of 95°C denaturation for 20 s, 56°C annealing for 30 s, and 72°C extension for 30 s, with a final 72°C incubation for 4 min. Dephosphorylation of unincorporated dNTPs was performed by adding 1.7 µl of H_2_O and 0.3 units of shrimp alkaline phosphatase (Sequenom), followed by incubation at 37°C for 20 min and then at 85°C for 10 min to deactivate the enzyme. Next, *in vivo* transcription and RNA cleavage were achieved by adding 2 µl of the PCR product to 5 µl of the transcription/cleavage reaction and incubation at 37°C for 3 h. The transcription/cleavage reaction contained 27 units of T7 R&DNA polymerase (EpiCentre, Palmerston North, New Zealand), 0.64× of T7 R&DNA polymerase buffer, 0.22 µl T Cleavage Mix (Sequenom), 3.14 mM DTT, 3.21 µl H_2_O, and 0.09 mg/ml RNaseA (Sequenom). Reactions were additionally diluted with 27 µl of H_2_O and conditioned with 6 mg of CLEAN Resin (Sequenom) for optimal mass-spectrum analysis. Samples were then dispensed with the MassARRAY nanodispenser (Samsung, Irvine, CA, USA) on a 384-well SpectroChip (Sequenom). Mass spectra were acquired using a MassARRAY Compact MALDI-TOF (Sequenom), and the methylation ratios were calculated by comparing the difference in spectra intensity derived from methylated and non-methylated template DNA by the Epityper software version 1.0 (Sequenom).

### Bisulfite sequencing

Genomic DNA (500 ng) was bisulfite converted with the EZ DNA methylation-Gold kit (D5005; Zymo Research, Orange, CA, USA) according to the manufacturer's recommendations. Modified DNA was amplified with the primers listed in Table S1. The PCRs were carried out with 25 ng bisulfite-treated DNA, 1 µl of HotStart *Taq* DNA polymerase (Qiagen), 10× supplied HotStart buffer, 4 µl of dNTP (25 mM), and 3 µl of PCR primers (5 µM). PCR conditions were 94°C for 15 min, then 40 cycles of 94°C for 30 s, 57°C for 30 s, and 72°C for 30 s. The PCR products were subjected to purification using the PCR cleanup kit (28160; Qiagen) following the manufacturer's instructions. The purified PCR products (150 ng) were then subcloned into a TA cloning vector (50 ng) (pGEM-T Easy vector; Promega, Madison, WI, USA). Several clones of each sample were verified by sequencing with the T7 universal primer.

### Chromatin immunoprecipitation (ChIP) and quantitative real-time PCR

ChIP was performed as previously described [32] with some modifications. Briefly, ChIP assays were carried out on 5×10^5^ NTera-2 and RNAi-treated cells. Cells were harvested by trypsinization, washed, and resuspended to 2×10^6^ cells/ml in PBS with 20 mM sodium butyrate. The protein-DNA complexes were fixed using 1% formaldehyde for 8 min, and crosslinking fixation was stopped by adding glycine to a final concentration of 125 mM for 5 min. Cross-linked cells were washed twice with cold PBS/20 mM butyrate, resuspended in 100 µl of lysis buffer (50 mM Tris-HCl at pH 8, 10 mM EDTA, 1% SDS, 1 mM PMSF, and 20 mM sodium butyrate supplemented with a fresh protease inhibitor cocktail (Sigma) and then sonicated to an average size of 250 bp by a MISONIX Sonicator 3000 for 30 min (with pulses of 30 s on and 30 s off). We used 5 µg of anti-Sin3A, 20 µg of anti-MeCP2 (sc-994X and sc-20700X; Santa Cruz Biotechnology), 5 µg of anti-STAT3 (05-485; Upstate-Millipore, New York, NY, USA), 5 µg of anti-CBP, 5 µg of anti-p300, 20 µg of anti-polII (sc-369X, sc-585X and sc-899X; Santa Cruz Biotechnology), 5 µg of anti-H3K9Ac, 5 µg of anti-H3K14Ac (06-942 and 06-911; Upstate-Millipore), and 2.5 µg of anti-H3K4me3 (ab8580; Abcam, Cambridge, MA, USA) for immunoprecipitation, which was performed at 4°C with the indicated antibodies by incubation with Protein A Dynabeads (Invitrogen) equilibrated with 1 ml RIPA buffer (10 mM Tris-HCl at pH 7.5, 1 mM EDTA, 0.5 mM EGTA 1% Triton X-100, 0.1% SDS, 0.1% Na-deoxycholate, and 140 mM NaCl) and protease inhibitors for 2 h. The immunocomplexes were further incubated with chromatin at 4°C overnight. The bound fraction was isolated by Protein A Dynabeads according to the manufacturer's instructions, and the immunoprecipitated complexes were eluted by using 300 µl of elution buffer (20 mM Tris-HCl at pH 7.5, 5 mM EDTA, 50 mM NaCl and 20 mM sodium butyrate) for 30 min at 65°C then for 10 min at room temperature on a rotator. Chromatin was eluted from the antibody and de-cross-linked by added of 30 µl pronase (20 mg/ml) and 1 µl CaCl_2_ (1 M) then incubating it at 42°C for 2 h and 65°C for 6 h. The immunoprecipitated DNA was recovered by a PCR purification kit (Qiagen) according to the manufacturer's instructions, and the purified samples were analyzed by real-time quantitative PCR using an SYBR Green master mix and a LightCycler 480 sequence detection system (Roche Applied Science, Mannheim, Germany). PCR primers were designed using the software program Primer Express (Roche Applied Science), and the amplification primers are listed in Table S2. For each sample, the PCR analysis was performed in triplicate, and the bound fraction was compared with 1∶40 diluted input DNA of 1×10^5^ cells. The results are reported as the ratio of immunoprecipitated (IP) DNA to input DNA (IP/input). To obtain relative occupancy values, the IP/input ratio was further normalized to the level observed at a control region, h, which was defined as 1.0. Values of NTera-2 cells were compared to those of RNAi-treated samples.

## Results

### Induction of differentiation of NTera-2 cells toward an astrocyte-like lineage

**Figure 1:**
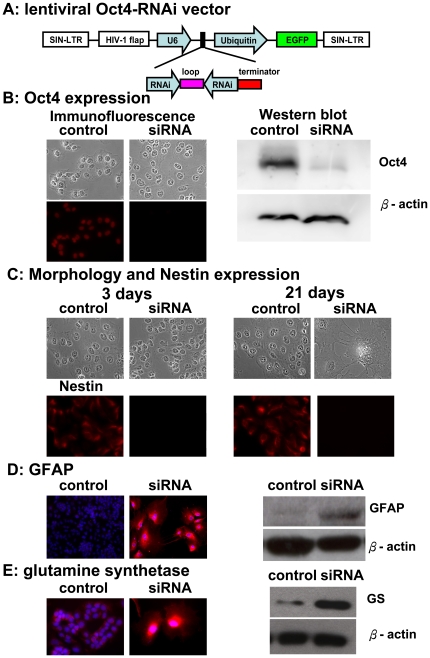
Differentiation of NTera-2 cells toward an astrocytic lineage. NTera-2 cells were induced to cellular differentiation by infection with lentiviruses engineered to express pFUGW-*Oct-4*-RNAi at a multiplicity of infection of 25. **A.** Construction map of pFUGW-*Oct-4*-RNAi. **B.** Immunofluorescence (red) and Western blots of Oct4 expression in undifferentiated NTera-2 cells and cells 21 days after differentiation. β-Actin was used as an internal control in the Western blots. Note the substantial reduction in Oct-4 expression after differentiation. **C.** Phase-contrast micrographs for the morphological analysis (upper panel) and immunofluorescence staining for nestin expression (lower panel, red) of NTera-2 cells 3 and 21 days after differentiation, respectively. Magnification 400×. **D.** Immunofluorescence (red) and Western blots for GFAP expression in undifferentiated NTera-2 cells and NTera-2 cells 21 days after differentiation. **E.** Immunofluorescence staining and Western blot analyses of glutamine synthetase (GS) expression in NTera-2 cells 21 days after differentiation. The red staining for GS was compared with Hoechst dye (blue) for nuclei. β-actin was used as an internal control in the Western blots.

NTera-2 cells are capable of differentiating into mixtures of neural and glial cells depending upon the induction conditions [33]–[35]. Since NTera-2 cells express high levels of Oct-4 [36], [37], we used a lentiviral vector carrying a short hairpin (sh)RNA (Fig. 1A) to downregulate Oct-4 expression in order to develop a new protocol for cell differentiation. Three days after transduction with the shRNA lentivirus, Oct-4 expression was greatly diminished (Fig. 1B). As shown in Fig. 1C, the cellular morphology remained the same as control NTera-2 cells at 3 days. However, dramatic morphological transformation was observed after 21 days of transduction, and cells had become flattened and enlarged, and showed a bushy morphology with numerous short and highly branched processes (Fig. 1C). Such a cell morphology exhibited characteristics similar to protoplasmic astrocytes, distinct from “fibrous astrocytes” [38]. In addition to these morphological changes, the reduction in Oct-4 expression by NTera-2 cells was accompanied by a marked reduction in cell proliferation (Fig. S1A). The growth potential of NTera-2 cells after transduction was studied by flow cytometry, and these cells had almost ceased cell cycling with accumulations of cells in the G0/G1 phase on days 4 and 14 (40.6% and 69.1%, respectively, in Fig. S1B). Thereafter, these cells remained mostly in the G0/G1 phase (e.g., on day 14) and underwent significant morphological changes, as described above. In addition, downregulation of Oct-4 expression often leads to upregulation of Cdx2 in ES cells [39], [40]. In NTera-2 cells, however, Cdx2 expression was not detected in RNAi- transduced cells (data not shown), indicating that this cell line may be derived from cells at a stage of embryogenesis later than the origin of the ES cell line. In addition, NTera-2 cells normally express the intermediate filament protein, nestin (Fig. 1C), a marker of neuroglial progenitor cells. But, the expression of nestin disappeared 3 days after transduction (Fig. 1C).

The morphological transformation and cessation of proliferation in RNAi-transduced cells suggested that NTera-2 cells might undergo a process of differentiation. To further verify that these changes were accompanied by differentiation toward an astrocyte-like lineage, cells were examined for the expression of astrocytic markers. The intermediate filament protein, GFAP, is considered a cell type-specific marker for astrocytes. GFAP expression was observed in differentiated cells examined by immunofluorescence and Western blot assays (Fig. 1D). Another astrocyte-specific marker, glutamine synthetase (GS) [41], was also detected by immunofluorescence and Western blot assays in differentiated cells (Fig. 1E). In addition, we detected neither the oligodendrocyte markers, A2B5 [42] and Gal-C [43], nor the neuron-specific markers, MAP-2 and Pax6 [44], after 21 days of transduction (Fig. S1C). Taken together, these differentiated NTera-2 cells produced by our newly developed protocol had an astrocyte-like morphology and expressed the astrocyte-specific markers, GFAP and GS, suggesting differentiation toward an astrocytic lineage. Therefore, these differentiated cells distinctly differed from previous reports of a mixture of different neuronal cell types after retinoic acid induction [35].

### Nuclear export of olig2 and STAT3 activation during cell differentiation

**Figure 2:**
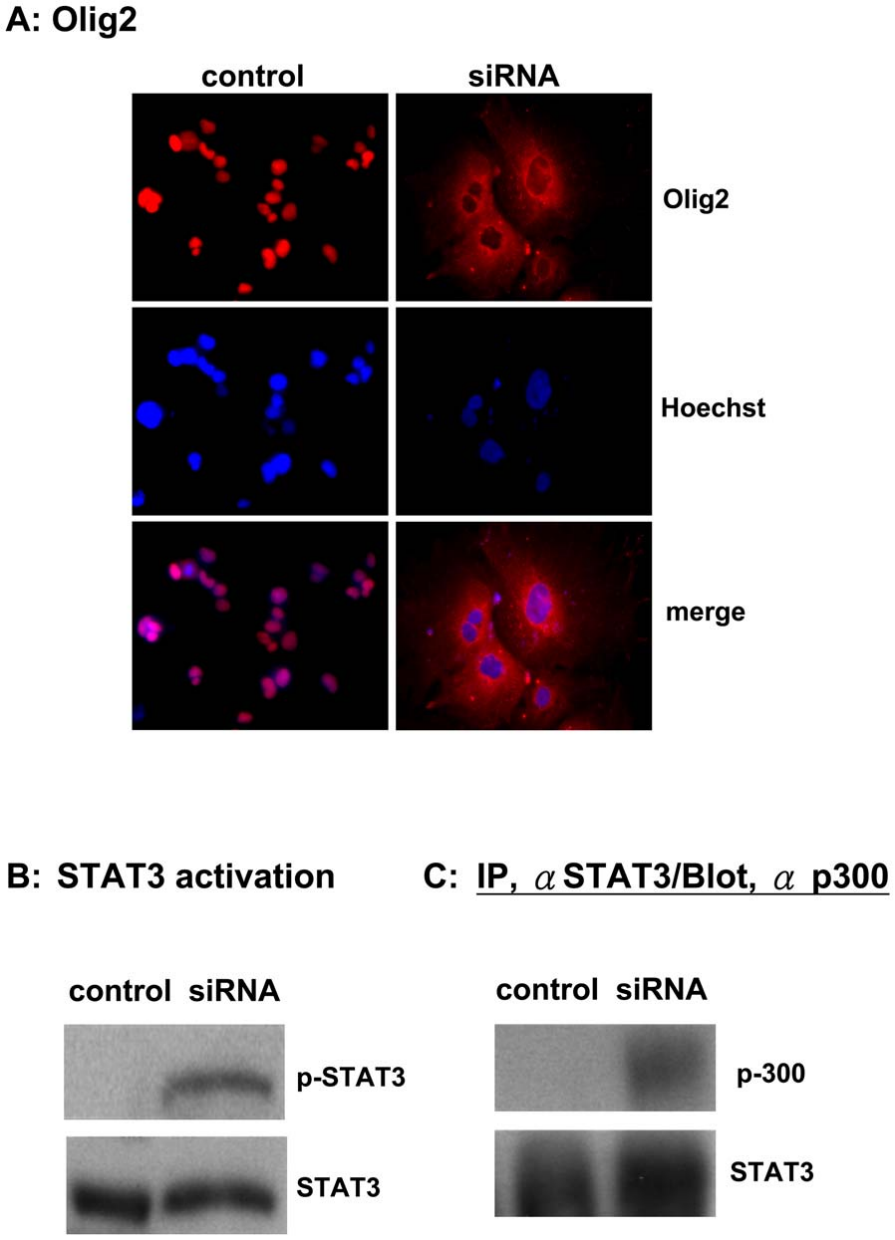
Translocation of olig2, activation of STAT3 signaling and STAT3-p300 complex formation, and loss of Sin3A after differentiation. A. Immunofluorescence staining for olig2 (red, upper panel), Hoechst dye (blue, middle panel), and merged images (bottom panel) are shown for undifferentiated control NTera-2 cells and NTera-2 cells 21 days after differentiation. Magnification 400×. B. STAT3 activation was examined with a specific antibody against phosphorylated STAT3, and total levels of STAT3 in lysates from undifferentiated NTera-2 cells and NTera-2 cells 21 days after differentiation (left panel) were determined. C. Lysates from cells similarly transduced were subjected to immunoprecipitation with an antibody against STAT3 and then were also probed with an anti-p300-specific antibody (right panel).

Oligo 2 is a basic HLH transcription factor, and its expression in the nucleus is required for oligodendrocyte development by preventing the formation of the STAT3 and p300 complex in the nucleus [45], [46]. To confirm the cellular differentiation of NTera-2 cells, we examined the expression of olig2 and found that olig2 was expressed in nuclei of undifferentiated NTera-2 cells (Fig. 2A). During specification for an astrocyte-like lineage, there was a striking translocation in the distribution of olig2 from nuclei to the cytoplasm (Fig. 2A). Thus, the translocation of olig2 from nuclei may suggest activation of STAT3 in nuclei. Therefore, we analyzed the phosphorylation status of STAT3 and found that there was significant accumulation of phosphorylated STAT3 in differentiated cells compared to undifferentiated cells (Fig. 2B). Furthermore, specific antibodies against STAT3 were found to have co-immunoprecipitated p300 in differentiated cells, but not in undifferentiated cells, suggesting specific formation of STAT3-p300 complexes in differentiated cells (Fig. 2C). These findings suggest activation of the STAT3 signaling pathway during the differentiation of NTera-2 cells.

### Reduction of histone co-repressor complexes during cell differentiation

**Figure 3:**
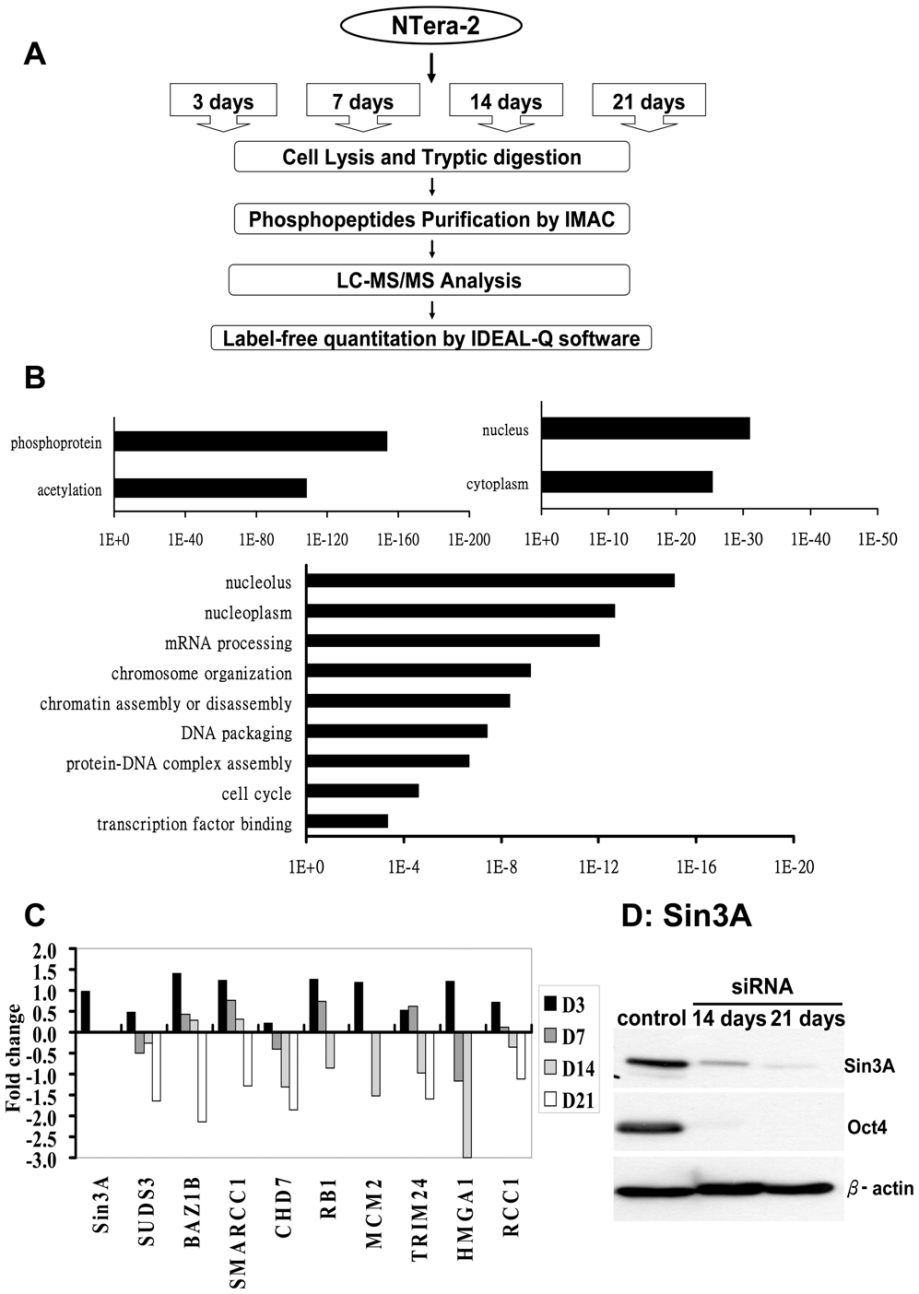
Phosphoproteomic signature in cellular differentiation revealed by a label-free quantitation strategy. **A.** Experiment workflow for quantitation of astrocyte phosphoproteomics. **B.** Functional annotation chart of the differentially phosphorylated proteins analyzed by DAVID [47], [48]; the highly represented categories are shown. Ontology terms are shown on the *y* axis; p-values for the significance of enrichment are graphed along the *x* axis. **C.** Analysis of the regulation of genes through label-free quantitation in NTera-2 cells 3, 7, 14 and 21 days during cell differentiation compared to undifferentiated NTera-2 cells. Data are expressed as log2 of fold change. **D.** Western blots showing levels of Sin3A expression in undifferentiated NTera-2 cells and NTera-2 cells 14 and 21 days after differentiation. β-actin was used as an internal control in the Western blots.

In order to investigate the mechanism of suppression and de-suppression of astrocyte differentiation-related genes in NTera-2 cells, we investigated and quantified differentially phosphorylated proteins using a newly developed label-free quantitation technology platform [30]. Briefly, phosphoproteins were digested in an optimized gel-assisted digestion and purified by a highly specific and reproducible automatic IMAC nanotechnology, followed by LC-MS/MS analysis. The relative quantification method was performed by software “IDEAL-Q” which was developed by SEMI alignment approach (Fig. 3A).

Using NTera-2, it was found that the dynamic phosphoproteomic profile was observed after induction toward astrocyte differentiation. We found that there was a slightly increased in the overall phosphoproteomic profile levels at day 14 after induction for differentiation. In contrast, the upregulation and downregulation levels of phosphoproteomic profile increased dramatically and more than 50% phosphorylation sites have differential levels after stimulation for 21days, suggesting the dramatic change in phosphoproteomic pattern.

To gain insight into the putative biological functions of the identified proteins, differentially phosphorylated proteins were functionally annotated and classified by using the functional annotation tool of Database for Annotation, Visualization and Integrated Discovery (DAVID) [47], [48] bioinformatics resources (http://david.abcc.ncifcrf.gov) on the National Institute of Allergy and Infectious Disease (NIAID). This software performed a statistical analysis based on gene ontologies to identify groups that were significantly overrepresented (enriched) in this filtered data set. Upon DAVID gene functional classification, there were two groups of annotated proteins identified that showed statistically significant enrichment scores; they included phosphoproteins and those that were posttranslationally modified by acetylation (Fig. 3B). In addition, these proteins with significant enrichment scores were separated to nucleus and cytoplasm in location. And the proteins that function in the nucleus were remarkably represented by those that control mRNA processing, chromosome organization, chromatin assembly/disassembly, DNA packaging, etc, (Fig. 3B).

Among several potential chromatin related functions, we chose to study epigenetic regulation and chromatin remodeling during cellular differentiation in more depth. Three important themes emerge from the shift in the expression of several known, classifiable proteins (Fig. 3C). First, the data set contains histone modifying complexes such as Sin3A and SUDS3. Second group of proteins are the components of chromatin remodeling complexes including BAZ1B, SMARCC1 and CHD7. They are thought to regulate chromatin structures using ATPase activity. The third is a group of chromatin associating proteins, such that RB1, MCM2, TRIM24, HMGA1 and RCC1. They are involved in diverse functions such as transcriptional control, DNA replication and chromosome condensation.

Of particular interest is the significant reduction of Sin3A in the phosphoproteomic pattern after 3 days of transduction. Sin3A is a component of Sin3A/HDAC co-repressor complexes, which functions in transcription repression [49]–[51], but its role in astrocyte differentiation has not been characterized. By a Western blot analysis of samples through the course of differentiation (control, 7, 14, and 21 days), we further confirmed that the protein expression of Sin3A decreased significantly and continuously after differentiation (Fig. 3D). Reduction of the expression of Sin3A may lead to disintegration of Sin3A/HDAC complexes thus causing de-suppression of the transcriptional program that favors astrocyte-like differentiation, such as activation of GFAP.

### Locus-specific reduction in Sin3A and MeCP2 occupancy

**Figure 4:**
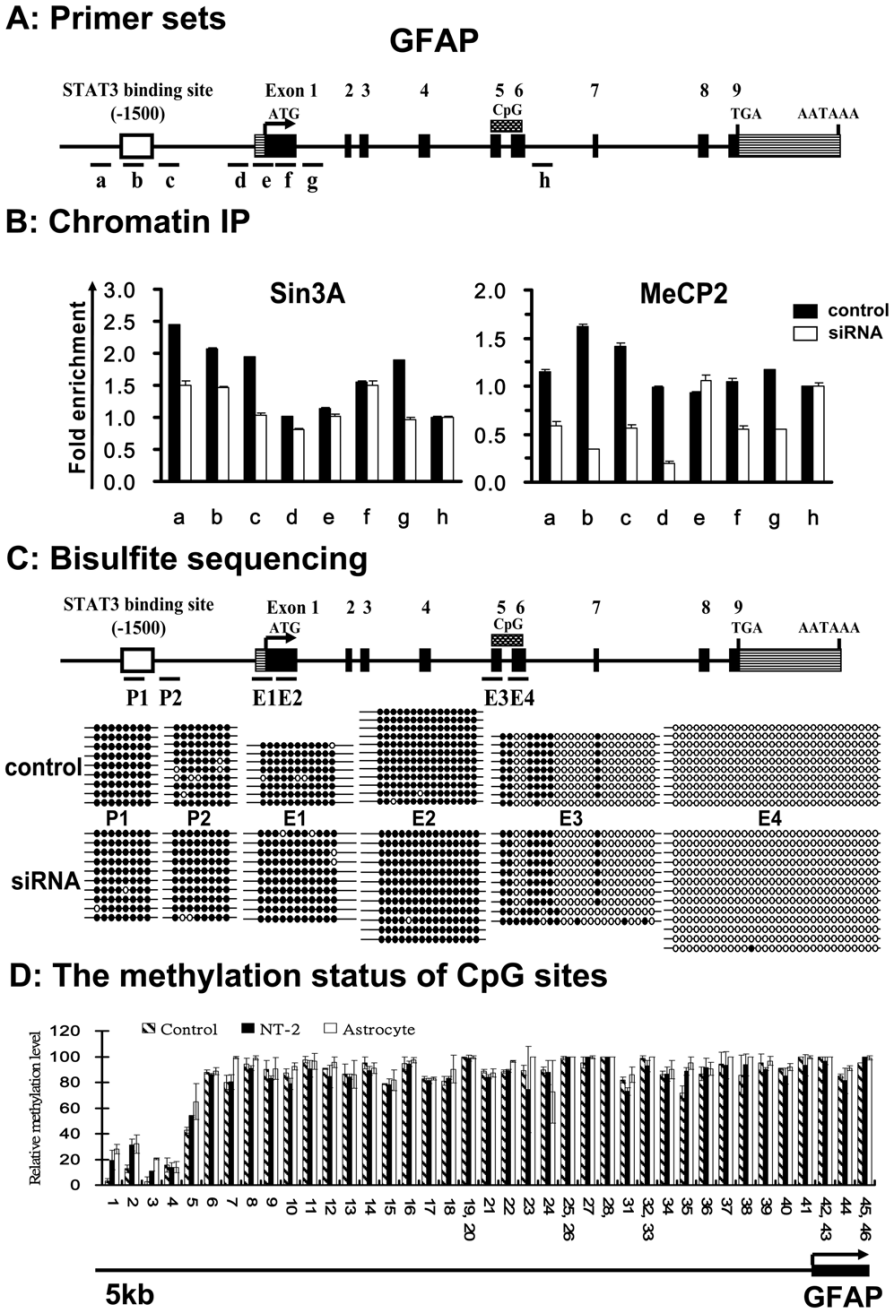
Sin3A and MeCP2 co-occupy the promoter of GFAP before differentiation. **A.** Schematic illustration of the structure of the proximal promoter and coding regions of GFAP. Black boxes indicate exons numbered with roman numbers. Lines connecting the exons are introns. Hatched boxes denote the 5′-untranslated region (UTR) and 3′-UTR. The start and stop codons are also indicated. qPCR primer pairs amplifying the STAT3-binding site and exon 1 regions are indicated as alphabetic letters. Region 3′ to the CpG island, h, served as a control primer pair. **B.** ChIP-qPCR analyses showing quantitative occupancies of Sin3A (left panel) and MeCP2 (right panel) in indicated regions of the GFAP gene in undifferentiated NTera-2 cells and at 21 days after differentiation. Multiples of enrichment are the relative abundances of the indicated regions over the control region, h. Error bars represent the means of triplicate values, and the standard deviation of one ChIP-qPCR experiment representative of two is shown. **C.** The methylation status of CpG sites within the STAT3 recognition sequence and GFAP gene exon 1 regions in undifferentiated NTera-2 cells and NTera-2 cells 21 days after differentiation was analyzed by bisulfite sequencing. Closed and open circles respectively indicate methylated and unmethylated CpG sites. **D.** Assays were performed on genomic DNA isolated from control, healthy individual blood samples. NT-2, undifferentiated NT-2 cells; astrocyte, astrocyte-like cells differentiated from NT-2 cells. The relative methylation levels of the indicated CpG sites (presented as a black bar) for the −5,000 bp upstream promoter were determined by a T-reverse cleavage reaction and MALDI-TOF/MS assay (Sequenom EpiTYPER platform).

Since there was a significant reduction in the expression of Sin3A at the cellular level after cell differentiation in NTera-2 cells, we next investigated regional alterations in chromatin around the STAT3-binding site at the promoter and transcription initiation sites of GFAP before and after astrocyte-like differentiation. To ascertain local changes in transcription factor binding, we performed ChIP assays with specific antibodies against Sin3A and MeCP2. In addition, the binding of these two co-repressors was quantitated by a real-time PCR using primers spanning from the STAT3 binding site at the promoter to exon 1 relative to the transcription start site of GFAP (primer sets a–g in Fig. 4A). In addition, a pair of primer, h (Fig. 4A), located between exons 6 and 7 near the end of GFAP gene and remote from the transcription regulatory region, served as a negative control. It was found that upon cellular differentiation, the binding of Sin3A significantly and specifically diminished around the STAT3 binding site about 1500 bp upstream of the GFAP start site in the promoter region (primer sets a–c in Fig. 4A) (Fig. 4B). In addition, we also identified that Sin3A occupancy at primer set g in the vicinity of exon 1 was strongly reduced after cell differentiation, whereas binding levels of Sin3A across the exon 1 locus (primer sets e and f) were not altered. Since Sin3A lacks an intrinsic DNA-binding capacity, it must target itself to promoters via interactions with other DNA-binding or adaptor proteins, such as MeCP2, a methyl-CpG-binding protein, which was reported to interact with Sin3A in *Xenopus laevis* oocytes [52]. To test the possibility that MeCP2 may target Sin3A to the GFAP promoter, we performed a ChIP analysis to inspect the binding of MeCP2 around the STAT3-binding site at the GFAP promoter and in the proximity of exon 1 before and after cell differentiation. As shown in Fig. 4B, the level of specific binding between MeCP2 and the STAT3-binding site at the GFAP promoter (primer sets a–c) and in the proximity of exon 1 (primer sets d–g, except site e) regions of GFAP in undifferentiated NTera-2 cells was substantially reduced in differentiated astrocyte-like cells. As the binding patterns for MeCP2 and Sin3A at the GFAP promoter and exon 1 occurred in a coordinated manner, these results suggest that MeCP2 may be responsible for recruiting the Sin3A co-repressor complex to the area surrounding the GFAP promoter.

MeCP2 is capable of binding to methylated DNA. There is a possibility that the reduction in MeCP2 occupancy at the GFAP promoter was triggered by DNA demethylation of the same region; we therefore examined the DNA methylation status of the GFAP gene in NTera-2 cells and differentiated astrocyte-like cells. However, the bisulfite sequencing analysis showed that upon differentiation toward an astrocyte-like lineage, the CpG dinucleotide around the STAT3-binding site at the promoter (primer sets P1 and P2 in Fig. 4C) and exon 1 (primer sets E1 and E2) was not demethylated (Fig. 4C). To further confirm these findings, we performed a quantitative methylation analysis using MALDI-TOF/MS to examine the mass signal shift of methylated DNA compared to unmethylated DNA extracted from undifferentiated NTera-2 cells, differentiated cells, and control human monocytes. This study confirmed that CpG sites scattered in the approximately 4500-bp region upstream of the GFAP transcription start site were stably methylated before and after differentiation (Fig. 4D). We concluded that the DNA methylation status of the GFAP promoter and exon 1 remained unchanged during cellular differentiation. Our results showed that reduced expression of Sin3A is accompanied by decreased occupancy of Sin3A and MeCP2 around the STAT3-binding site of the GFAP promoter, which was not necessarily accompanied by DNA demethylation in the same region for the induction of GFAP expression. These results suggest a critical role for Sin3A in regulating GFAP expression during astrocytic differentiation.

### Activation of GFAP expression

**Figure 5:**
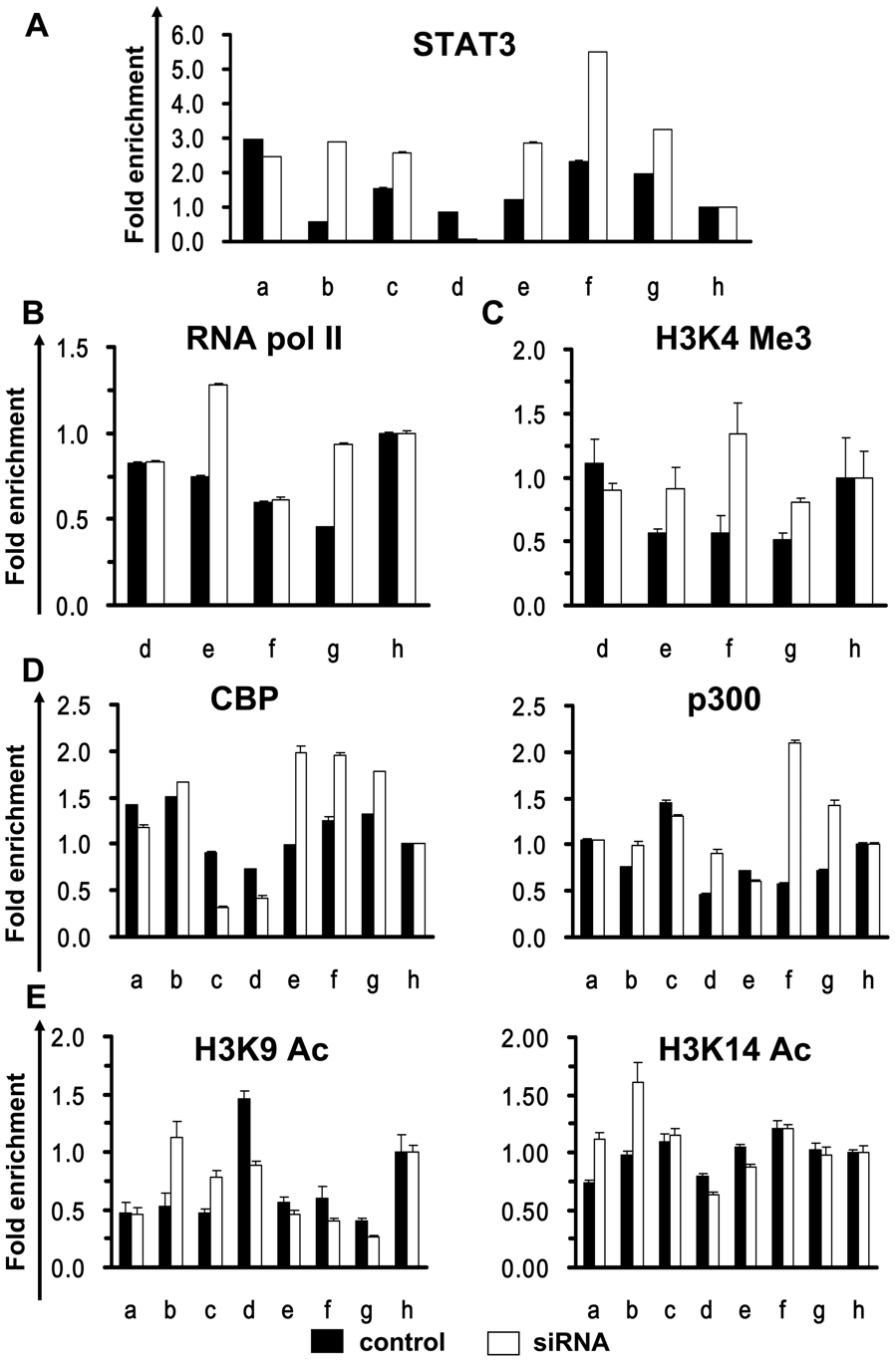
STAT3 occupies both the promoter and exon 1 of GFAP, recruitment of CBP and p300 to exon 1, and changes in histone acetylation levels at the STAT3-binding site after differentiation. ChIP analyses were performed using anti-STAT3-P (A), RNA polymerase II (B), and H3K4me3 (C) antibodies and qPCR primer pairs (Fig. 4A) to detect the indicated regions of the GFAP gene in undifferentiated NTera-2 cells and cells 21 days after differentiation. D. Chromatin samples from undifferentiated NTera-2 cells and cells 21 days after differentiation were immunoprecipitated with anti-CBP (left panel) and anti-p300 (right panel) antibodies, and enrichment was quantitated by qPCR. E. A ChIP assay was performed as described in panel D but with active histone modifications, and anti-H3K9Ac (left panel) and anti-H3K14Ac (right panel) antibodies. Multiples of enrichment are the relative abundances of the indicated DNA fragments over the control fragment, h. Error bars represent the means of triplicate values, and the standard deviation of one ChIP-qPCR experiment representative of two is shown.

The binding of activated STAT3 to a STAT3 recognition element (−1518 to −1510 bp in relation to the transcription start site) in the mouse GFAP promoter plays a major role in the transcriptional activation of GFAP [53]. Activation of STAT3 was also identified in astrocyte-like cells (Fig. 2B), which suggested that STAT3 may be responsible for GFAP activation. To test this, the occupancy of activated STAT3 was examined by a ChIP assay, using similar primer sets a–g as shown in Fig. 4A. As anticipated, the occupancy of activated STAT3 was more abundant around the STAT3- binding site at the GFAP promoter (primer sets b and c, especially primer set b at the STAT3 binding site) in differentiated cells compared to undifferentiated cells (Fig. 5A). Interestingly, at the exon 1 coding region and the 3′ end of the exon 1 region of the GFAP gene, we also detected a second peak of activated STAT3 binding signals (primer sets e–g) in differentiated cells but not in undifferentiated NTera-2 cells (Fig. 5A). STAT3 is a sequence-specific DNA-binding protein; in addition to the binding site at −1500 bp in the promoter (75% identities), we had identified a second binding site at +250 bp (TTCCTGGAA) at exon 1 of human GFAP (85% identities), based on a detailed sequence comparison with mouse GFAP gene. These results argue that STAT3 may originally target the binding sequence that resides at the GFAP promoter, but due to the chromatin configuration between the promoter and exon 1 in differentiated cells, STAT3 is also cross-linked to chromatin at exon 1. Alternatively, the occupancy of activated STAT3 may cause a bridge between the promoter and exon 1 which induces a stereospecific interaction on surfaces around the GFAP promoter and transcription start site.

To further validate activation of GFAP induced by STAT3 binding, a ChIP analysis was performed to examine the recruitment of RNA polymerase II. A comparison of undifferentiated NTera-2 cells with differentiated cells revealed increased recruitment of RNA polymerase II to the GFAP gene transcription start site (primer set e) after differentiation, consistent with enhanced transcription (Fig. 5B). Previous studies suggested that H3 Lys4 trimethylated (H3K4me3) nucleosomes occur near transcription start sites of actively transcribed genes. Moreover, several large-scale and genome-wide analyses revealed that about 91% of all RNA polymerase II-binding sites are correlated with H3K4me3-binding sites and were positively correlated with gene expression levels [54], [55]. Using a ChIP assay, we also found that H3K4me3 was enriched at sites of transcription initiation (primer sets e–g), with maximal enrichment immediately downstream of the start site (primer sets e and f) and overlapping the peak of RNA polymerase II at the transcription start site (primer set e) in differentiated cells compared to undifferentiated cells (Fig. 5C). These results suggest a close correlation among STAT3-mediated chromatin remodeling, RNA polymerase II recruitment, and GFAP activation.

Figure 2B and 2C show that activated STAT3 was co-immunoprecipitated with p300. Furthermore, it was reported that STAT proteins activate transcription by recruiting the transcription co-activator, CBP/p300 [56], [57], and CBP/p300-induced acetylation on histone tails is coupled with chromatin remodeling, thereby enhancing target gene expression [58], [59]. We next examined whether the CBP/p300 co-activator was also associated with the activated GFAP promoter along with an increase in the histone acetylation level in the same region. Notably, we did not detect a significant association between CBP/p300 proteins and the STAT3-binding site at the GFAP promoter (primer sets a–c), as indicated by the ChIP analyses (Fig. 5D). Conversely, upon cellular differentiation, robust recruitment of CBP/p300 proteins was observed in the vicinity of the exon 1 coding region (primer sets e–g with the CBP antibody and primer sets d, f, and g with the p300 antibody) (Fig. 5D). Since there is no report of DNA-binding ability of CBP/p300 in these regions, this result suggested that CBP/p300 was brought to the vicinity of exon 1 by transcription factors such as STAT3 (Fig. 5A) after cell differentiation. We further inspected the status of histone acetylation before and after differentiation to survey the influence of CBP/p300 deposition on exon 1. ChIP assays were conducted with specific antibodies against acetyl-H3K9 (H3K9Ac) and acetyl-H3K14 (H3K14Ac) in undifferentiated NTera-2 cells and differentiated cells. The results showed that there were significant increases in active markers of H3K9Ac (primer sets b and c) and H3K14Ac (primer sets a and b) around the STAT3-binding site at the GFAP promoter region, particularly the STAT3-binding site (primer set b), but not in the exon 1 coding region (Fig. 5E). In addition, in a separate experiment (Liao, CH and Yu, J. unpublished observation), we found that treatment of NTera-2 cells with HDAC inhibitors led to an increase in the acetylated H3K9 and H3K14 using specific antibodies and also GFAP gene expression. Therefore, our results raise the possibility that the chromatin structure of the GFAP promoter may undergo dramatic changes in organization during cell differentiation.

## Discussion

### Reduction of Sin3A during cell differentiation

Astrocytes are the most numerous cells in the CNS that provide important support to neurons and modulate synaptic activity [60]. In the present study, we developed a new protocol to obtain pure population of astrocyte-like cells from human NTera-2. We revealed a novel role for Sin3A which coupled to MeCP2 and bound to the STAT3-binding site in the GFAP promoter and its occupancy was inversely correlated with de-suppression of GFAP transcription. Sin3A is thought to be devoid of intrinsic DNA- binding capacity, but is able to be recruited by MeCP2 that linked to methylated DNA with HDAC complex. Such formation of corepressor complex by chromatin-bound MeCP2 may lead to local deacetylation of core histones with subsequent transcriptional silencing [52], [61]. Recent reports proposed that another corepressor, N-CoR, controls differentiation of neural stem cells into astrocytes [62] because the complex containing N-CoR, Sin3A and HDAC mediated transcriptional repression [63], [64]. In fact, our phosphoproteomic analysis also confirmed the presence of N-CoR2 in the undifferentiated NTera-2, which declined significantly after cell differentiation (data not shown). These results support transcriptional repression by Sin3A/MeCP2 complex serving as one of the critical mechanisms underlying the inhibition of astrocytic differentiation. It seems that MeCP2 with Sin3A binds to the methylated STAT3-binding site of the GFAP promoter, thus making the site inaccessible to STAT3. Upon cellular differentiation, occupancy by MeCP2 and Sin3A was significantly and specifically diminished at the STAT3-binding site of the GFAP promoter, suggesting a new regulatory path to trigger GFAP activation, in addition to the regulation by STAT3.

### Conformational change in the GFAP promoter during cell differentiation

**Figure 6:**
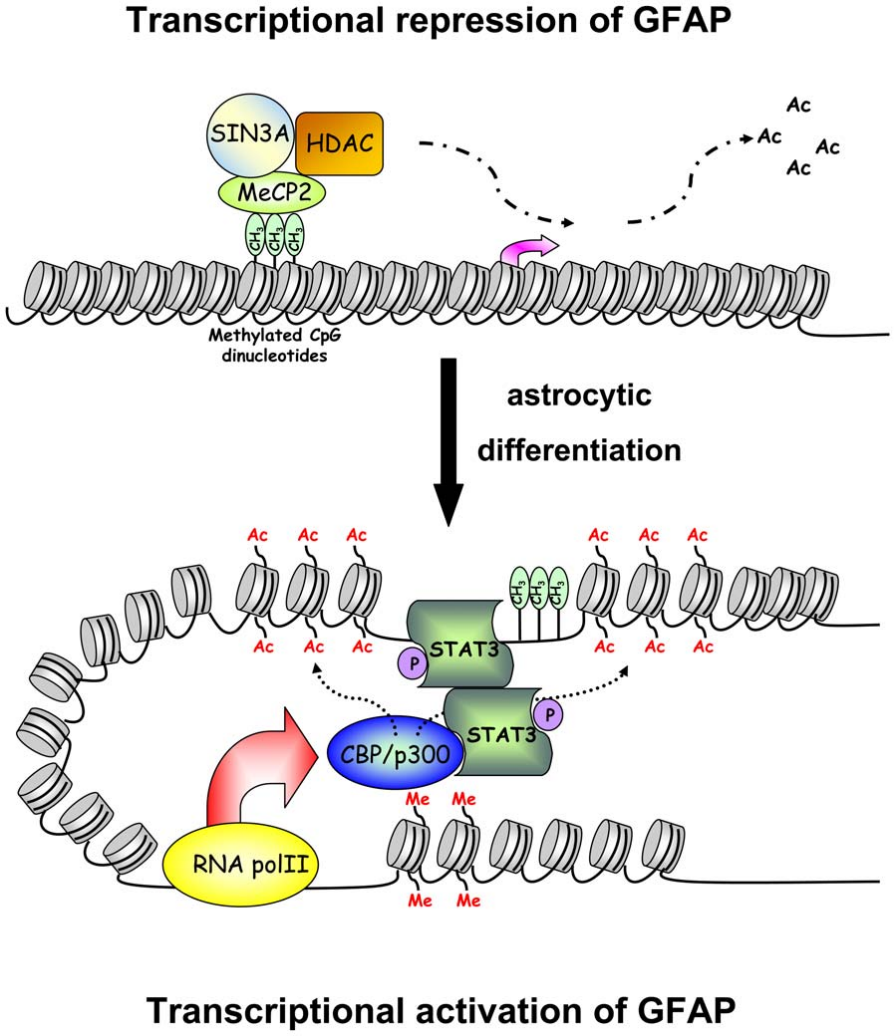
Model for GFAP gene activation for induced human astrocytic differentiation. In undifferentiated NTera-2 cells, the presence of the MeCP2 and Sin3A corepressor complex and possibly associated HDAC activity at the GFAP promoter maintain the deacetylated status of chromatin and repress GFAP transcription. Upon cellular differentiation, MeCP2 and Sin3A became dissociated from the GFAP promoter, and the subsequent recruitment of the phosphorylated STAT3 caused a conformational change in the region surrounding the GFAP transcription starting site. This, in turn, facilitated histone H3 acetylation of the promoter resulting from the recruitment of CBP/p300 to exon 1. The combination of chromatin remodeling and promoter conformational changes enabled the recruitment of RNA pol II.

On the other hand, in differentiated cells, activated STAT3, present at both STAT3-binding sites of the GFAP promoter and its exon 1 suggests that a conformational change may bridge both sides of the transcriptional start site during GFAP activation. This conformational change resulted in subsequent recruitment of CBP/p300 to the exon 1 coding region but not the promoter in the ChIP assay. Finally the specific increase of CBP/p300 occupancy at GFAP locus implies that the acetylation by these histone acetyltransferases play roles for gene activation of GFAP. Furthermore, CBP/p300 targeted histone H3 acetylation of the promoter but not exon 1 and induced chromatin remodeling, thereby enhancing recruitment of RNA polymerase II to activate GFAP transcription (Fig. 6). In addition, this notion of the change of the acetylation status accompanied with GFAP expression in NTera-2 cells was also supported by another independent assay in which specific HDAC inhibitors were used to increase the acetylated H3K9 and H3K14 in NTera-2 cells (Liao, CH and Yu, J., unpublished observation).

Processes that regulate gene transcription are directly under the influence of genome organization, and intrachromosomal interactions such as chromatin looping were shown to be involved in promoting transcriptional activation of genes in eukaryotes [65], [66]. Our ChIP experiments showed that activated STAT3 was present both in STAT3-binding sites of the promoter and in the exon 1 coding region of GFAP gene, and CBP/p300 was specifically recruited to the exon 1 coding region but not to the promoter. These findings imply that there are conformational changes in the GFAP brought about by STAT3 activation.

To investigate whether the interaction between STAT3 and CBP/p300 can result in the formation of a DNA loop between the promoter and coding region of GFAP, a 3C (chromosome conformation capture) assay [67] was performed. We also combined the 3C with ChIP (using p300 as a ChIP antibody) in a ChIP-loop assay [68] that presumably allowed us to determine which genomic sites would interact and how the candidate proteins mediate the interactions. Unfortunately, we failed to detect direct evidence of DNA looping at the GFAP promoter and thus conclusions must await further study. Although we had not yet establish a cause-and-effect relationship between DNA looping and GFAP gene transcription, our data of STAT3 and CBP/p300 binding suggest the possibility of DNA looping in the region surrounding the GFAP promoter. Alternatively, it was shown that during cell differentiation from a pluripotent to a committed state, there were global changes in the chromatin structure and interactions of chromatin-binding proteins [69]. Therefore, we cannot exclude the possibility that intrachromosomal interactions occurred in other regions where we did not examine in this study or that interactions between the promoter and coding region of GFAP may still occur in a more complicated manner [70].

### Activation of GFAP without DNA demethylation

It is known that binding of activated STAT3 to a consensus site in the GFAP promoter plays a role in the transcriptional activation of GFAP [53]. Our observations showed that GFAP expression was induced during astrocyte-like differentiation of NTera-2. ChIP experiments confirmed that there was a strong association of STAT3 with the GFAP promoter, suggesting the existence of mechanism that facilitates access of the STAT3 complex to the GFAP promoter. Methylation of CpGs in DNA constitutes one epigenetic mark that generally correlated with transcriptionally silent chromatin, and hypomethylated DNA in the promoter is a hallmark of vertebrate genes that are actively transcribed [71]. In mouse model, GFAP activation was associated with the loss of DNA methylation at the STAT3-binding site [22]. However, our investigation on the epigenetic regulation of GFAP expression in human NTera-2 cells did not observe changes of DNA methylation at the promoter (up to 4,500 bp upstream), indicating that regulation of the interaction of the STAT3 complex in the GFAP promoter was not mediated by DNA demethylation [21]. There seem to be several explanations that may account for the discrepancies between our results and previous data from the mouse study. First, since no enzyme has yet been identified that actively removes the methyl group from DNA, it is believed that DNA methylation is passively removed through multiple rounds of DNA replication [72]. In present study, the differentiated cells from NTera-2 underwent growth arrest on day 3 after induction. Therefore, it was not possible for these cells to have reduced the methylation level of DNA through cell division. Secondly, it was recently observed that DNA hypermethylation was completely maintained at the promoter region of the erythroid- specific carbonic anhydrase II upon hormone-induced activation [73]. Thus this finding challenges the paradigm that the methylation of promoter-containing CpG islands invariantly causes gene silencing. Finally, it was reported that parental allele-specific histone modifications in the promoter, rather than the differentiated methylated DNA, marked the imprinting status of imprinted genes [74].

In other word, the promoter-restricted change of histone modifications is one of the governing epigenetic marks in transcriptional regulation. Studies in yeast have demonstrated that nucleosomes with histone H3K4me3 are associated with actively transcribed genes [75]–[80]. Similarly, acetylation of histone H3K9 and H3K14 is critical for the recruitment of transcription factor II D, an initiation step in transcription, and therefore be associated with actively transcribed genes [77], [79], [81]–[83]. Histone acetylation is catalyzed by several evolutionarily conserved histone acetyltransferases, including Gcn5/PCAF, TAF1, and CBP/p300 [84]. Genome-wide analysis in human cells confirmed that H3K4me3, H3K9Ac and H3K14Ac are present together at actively transcribed genes [85]. Our ChIP experiments showed that upon GFAP activation after cell differentiation, there were significant increases in active markers of H3K9Ac and H3K14Ac around the STAT3-binding site at the GFAP promoter region. At the same time, H3K4me3 was enriched and overlapped with the peak of RNA polymerase II at the transcription start site. These results support the notion that chromatin remodeling regulated by histone modification is linked to active transcription and provide evidence that the acetylation status of chromatin at the GFAP promoter is likely to be predominant regulator of transcription. Altogether, our study has added a novel regulatory path to GFAP gene expression in addition to DNA methylation.

### Induction of differentiation of NTera-2 cells toward an astrocyte-like lineage

Human NTera-2 cells are widely used as models in human neurogenesis and differentiate into mixtures of various neuronal cell types [35], [86]; but directed differentiation toward specific lineages such as astrocytic cells has not been accomplished. In this study, we developed an *in vitro* model of cellular differentiation in which human NTera-2 cells were differentiated into a homogenous population of cells with an astrocyte-like morphology and expressing astrocyte-specific, but not neural or oligodendrocyte markers. The advantages of this model over other *in vitro* systems for human astrocyte-like differentiation include its capability for robust differentiation and the formation of astrocyte-like cells but not other neuronal cell types. Such directed differentiation differs from those which induce differentiation into mixtures of mostly neurons and a few astrocytes after the 4- or 5-week treatment with retinoic acid [35]. Thus this *in vitro* human cell model can be used to examine a detailed mechanism underlying astrocytic differentiation. By now there is strong evidence that astrocyte development and regeneration play critical roles in repair after brain injury and future studies should clarify how these astrocytes are regenerated and how precursor cells can be manipulated to recover from brain injuries.

## Supporting Information

Figure S1 **Cell proliferation cessation and cell-cycle arrest induced during astrocytic differentiation.** **A.** Growth of NTera-2 cells before and after differentiation as analyzed with a hemocytometer (*n* = 3; error bars indicate standard deviations). **B.** For the cell-cycle analysis, NTera-2 cells were similarly induced and then collected, stained with propidium iodide, and analyzed by flow cytometry. The ratios of cells in the G0/G1 phase on days 4, 7, and 14 days are shown. **C.** Immunofluorescent localization of A2B5, Gal-c, MAP-2, and Pax6 was examined in undifferentiated NTera-2 cells and cells 21 days after differentiation. Magnification 400×.
(TIF)

Table S1 **List of primers used for bisulfite sequencing.**
(DOC)

Table S2 **List of primers used for chromatin immunoprecipitation and quantitative real-time PCR.**
(DOC)
