# Supplementary figures and images for: Interplay between SIN3A and STAT3 Mediates Chromatin Conformational Changes and GFAP Expression during Cellular Differentiation
Source: PLoS One. 2011 Jul 11;6(7):e22018. doi: 10.1371/journal.pone.0022018 (PMC3136934; doi:10.1371/journal.pone.0022018)

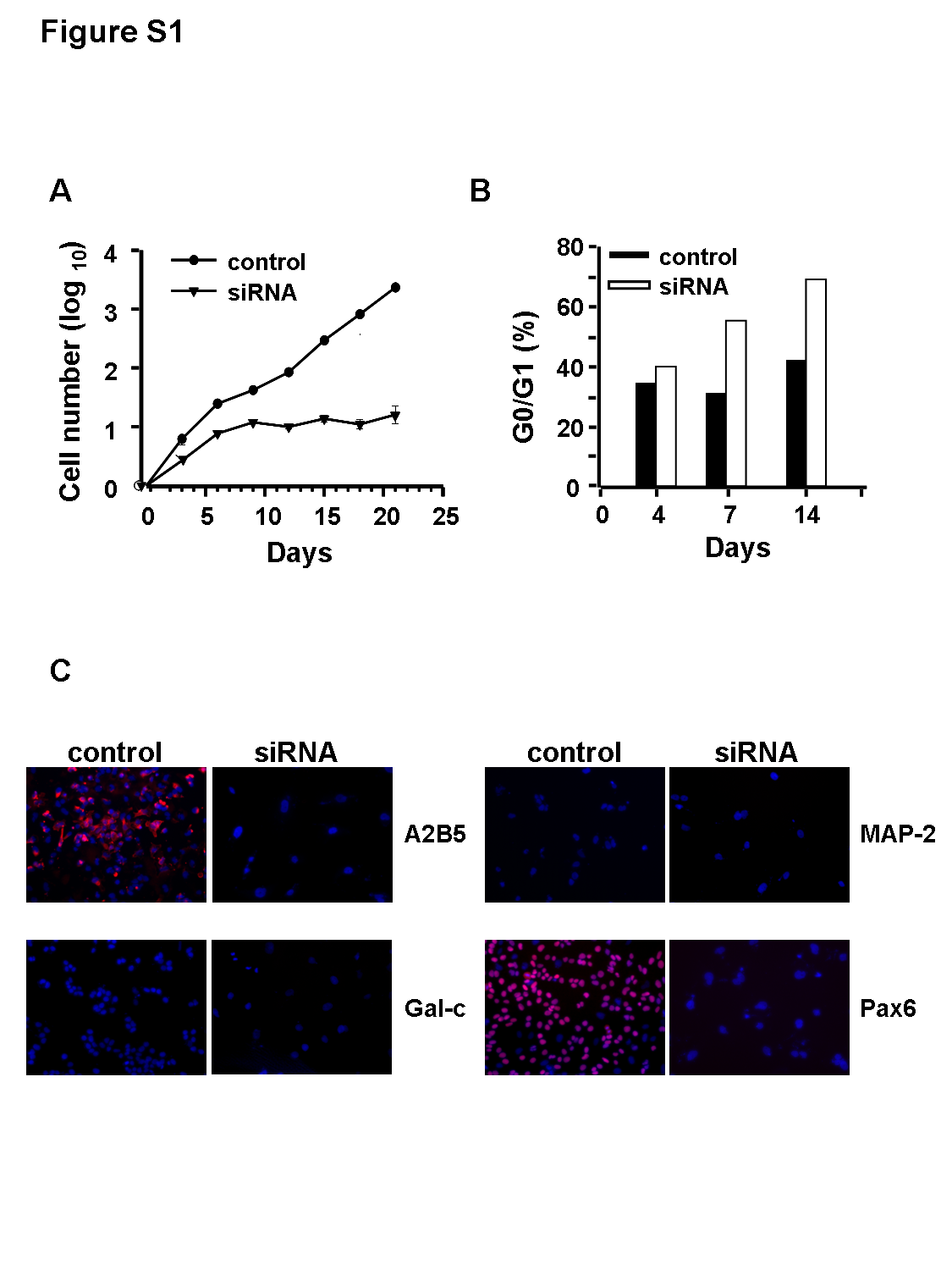

Supplement: Figure S1 — Cell proliferation cessation and cell-cycle arrest induced during astrocytic differentiation. A. Growth of NTera-2 cells before and after differentiation as analyzed with a hemocytometer (n = 3; error bars indicate standard deviations). B. For the cell-cycle analysis, NTera-2 cells were similarly induced and then collected, stained with propidium iodide, and analyzed by flow cytometry. The ratios of cells in the G0/G1 phase on days 4, 7, and 14 days are shown. C. Immunofluorescent localization of A2B5, Gal-c, MAP-2, and Pax6 was examined in undifferentiated NTera-2 cells and cells 21 days after differentiation. Magnification 400×. (TIF) [file pone.0022018.s001.tif]
