# Supplementary material for: Interplay between SIN3A and STAT3 Mediates Chromatin Conformational Changes and GFAP Expression during Cellular Differentiation
Source: PLoS One. 2011 Jul 11;6(7):e22018. doi: 10.1371/journal.pone.0022018 (PMC3136934; doi:10.1371/journal.pone.0022018)
Supplement: Table S1 — List of primers used for bisulfite sequencing. (DOC) [file pone.0022018.s002.doc]

**Table S1. Table S1 list of primers used for bisulfite sequencing.**

| Primer | Sequence |
| --- | --- |
| hGFAP-P1-F | AGGAGGGTTGTTTGTTTTTTAGAA |
| hGFAP-P1-R | CCCTTCCTTATCTAACCTCCCTATA |
| hGFAP-P2-F | GTAGATTTGGTAGTATTGGGTTGGT |
| hGFAP-P2-R | CCCTCACCCATTTATATCCTTAAA |
| hGFAP-E1-F | GTTGGGGTATTTAATGTTGGTTTTA |
| hGFAP-E1-R | TAAAAATTCAACCCCTTCTACTCAC |
| hGFAP-E2-F | TAGGTTATGTTAGGGGGTGTTGTTA |
| hGFAP-E2-R | ATCATTAAACTCCATCATCTCTACC |
| hGFAP-E3-F | GTGTTAGGTGTTGAGGTTTGGTAGT |
| hGFAP-E3-R | CTCCCCTAACATCTCCTAAAATAAC |
| hGFAP-E4-F | TTATTTTAGGAGATGTTAGGGGAGAA |
| hGFAP-E4-R | CAAAAAAACTTCCCCAAAAACTATA |
