# Supplementary material for: Interplay between SIN3A and STAT3 Mediates Chromatin Conformational Changes and GFAP Expression during Cellular Differentiation
Source: PLoS One. 2011 Jul 11;6(7):e22018. doi: 10.1371/journal.pone.0022018 (PMC3136934; doi:10.1371/journal.pone.0022018)
Supplement: Table S2 — List of primers used for chromatin immunoprecipitation and quantitative real-time PCR. (DOC) [file pone.0022018.s003.doc]

**Table S2. Table S2 list of primers used for chromatin immunoprecipitation and quantitative real-time PCR.**

| Primer | Sequence |
| --- | --- |
| hGFAP-a-F | cttacgcccaggtcagatgt |
| hGFAP-a-R | cctctgtgagtcccagcac |
| hGFAP-b-F | tccgagaagcccattgag |
| hGFAP-b-R | tgtgctgcttttatcccaaga |
| hGFAP-c-F | cagagtggagggcgtagatg |
| hGFAP-c-R | caactaggagcctgagacctg |
| hGFAP-d-F | agcccttccttcccttttt |
| hGFAP-d-R | ggctgaggctggaggatt |
| hGFAP-e-F | TCGCCAGTCTAGCCCAC |
| hGFAP-e-R | ATGCGTCTCCTCTCCAT |
| hGFAP-f-F | GCTTCCTGGAACAGCAAAAC |
| hGFAP-f-R | AGGTCCTGTGCCAGATTGTC |
| hGFAP-g-F | TTTGTGACTGTGGGCAGC |
| hGFAP-g-R | CCAGAATCCAATCTCCCTCAT |
| hGFAP-h-F | agccaagattggactcctca |
| hGFAP-h-F | gaggggaaagtggtgaagaa |
